# Prevalence of drinking or eating more than usual and associated factors during childhood diarrhea in East Africa: a multilevel analysis of recent demographic and health survey

**DOI:** 10.1186/s12887-022-03370-7

**Published:** 2022-05-23

**Authors:** Habitu Birhan Eshetu, Samrawit Mihret Fetene, Ever Siyoum Shewarega, Elsa Awoke Fentie, Desale Bihonegn Asmamaw, Rediet Eristu Teklu, Fantu Mamo Aragaw, Daniel Gashaneh Belay, Tewodros Getaneh Alemu, Wubshet Debebe Negash

**Affiliations:** 1 Department of Health Education and Behavioral Sciences, Institute of Public Health, College of Medicine and Health Sciences, University of Gondar, P.O. Box: 196, Gondar, Ethiopia; 2 Department of Health Systems and Policy, Institute of Public Health, College of Medicine and Health Sciences, University of Gondar, Gondar, Ethiopia; 3 Department of Reproductive Health, Institute of Public Health, College of Medicine and Health Sciences, University of Gondar, Gondar, Ethiopia; 4 Department of Epidemiology and Biostatistics, Institute of Public Health, College of Medicine and Health Sciences, University of Gondar, Gondar, Ethiopia; 5 Department of Human Anatomy, College of Medicine and Health Sciences, University of Gondar, Gondar, Ethiopia; 6 Department of Pediatrics and Child Health Nursing, School Of Nursing, College of Medicine and Health Sciences, University of Gondar, Gondar, Ethiopia

**Keywords:** Prevalence, Drinking or eating more, Children, East Africa

## Abstract

**Background:**

Diarrhea is the second most common cause of death in under-five children. Fluid and food replacement during diarrheal episodes have a paramount effect to avert morbidity and mortality. However, there is limited information about feeding practices. This study aimed to assess the prevalence of drinking or eating more and associated factors during diarrhea among under-five children in East Africa using demographic and health surveys (DHSs).

**Methods:**

Secondary data analysis was done on DHSs 2008 to 2018 in 12 East African Countries. Total weighted samples of 20,559 mothers with their under-five children were included. Data cleaning, coding, and analysis were performed using Stata 16. Multilevel binary logistic regression were performed to identify factors associated with drinking or eating more during diarrheal episodes. Adjusted Odds Ratio (AOR) with a 95% CI, and *p*-value < 0.05 were used to declare statistical significance.

**Results:**

Prevalence of drinking or eating more than usual during diarrhea disease in East Africa was 26.27%(95% CI: 25.68–26.88). Mothers age > 35 years (AOR: 1.14, 95% CI: (1.03, 1.26), mothers primary education (AOR: 1.17, 95% CI: 1.06,1.28), secondary education (AOR: 1.43,95% CI: 1.27,1.61), and higher education (AOR: 1.42,95% CI: 1.11,1.81), occupation of mothers (agriculture, AOR: 2.2, 95% CI: 1.3–3.6), sales and services, AOR = 1.20, CI:1.07,1.34), manual, AOR =1.28,95% CI: 1.11,1.44), children age 1–2 years (AOR =1.34,95% CI: 1.22,1.46) and 3–4 years (AOR =1.36,95% CI: 1.20,1.55), four and more antenatal visits (AOR: 1.14,95% CI: 1.03,1.27), rich wealth status (AOR:1.27,95% CI: 1.16,1.40), birth in health facility (AOR = 1.19, 95%CI: 1.10, 1.30) and visit health facility (AOR = 1.12, 95%CI: 1.03, 1.22) were associated with drinking or eating more.

**Conclusion:**

The prevalence of drinking or eating more is low in East Africa. Maternal age, occupation, antenatal care visit, marital status, educational status, wealth status, place of delivery, visiting health facility, and child age were significantly associated with drinking or eating more during diarrheal episodes. Health policy and programs should focus on educating mothers, improving the household wealth status, encouraging women to contact health facilities for better feeding practices of children during diarrheal episodes.

## Background

According to the world health organization (WHO), diarrhea is the passage of three or more loose or liquid stools per day or more frequent passage than normal for the child and is the second most killer, preceded by pneumonia, of children under the age of five [1, 2]. Every year approximately 1.7 billion cases of childhood diarrhea are detected and are responsible for the killing of around 525,000 children worldwide [1], of which 90% of death is in low and middle-income countries [3]. In Africa, diarrhea is the leading cause of mortality among young children, accounting for more than half of all deaths among children under the age of five, and globally, it is responsible for one in ten child deaths each year [4, 5]. Childhood diarrhea has a consequence on the child’s development and cognitive abilities [6]. Children who suffer from diarrhea during their first 2 years of life may have an 8 cm growth decrement and a 10 intelligent quotient point decrement when they reach 7–9 years old [5].

Dehydration is the commonest cause of death in children with diarrhea. Diarrhea-induced dehydration can also result in the loss of important nutrients, resulting in micronutrient deficits and severe malnutrition in children [7]. Although fluid replacement during diarrheal episodes can avert a large share of death from diarrheal illness, only a small proportion of children experiencing life-threatening episodes of diarrhea receive treatment [8, 9]. To avert diarrheal disease-related mortality and morbidity, WHO and UNICEF devised a seven-point action plan for comprehensive diarrhea control [10]. During a diarrhea attack, fluid replacement, continued feeding, and increasing suitable fluids in the home are indeed the mainstays of its treatment [11, 12]. About 39% of children under the age of five in developing countries and 34% of children under the age of five in Africa receive fluid replacement during diarrheal episodes [11, 12].

Diarrhea in children is usually connected with poor household features, such as a poor parental education [13, 14], a big family size [13], lower socio-economic status [14], and also maternal employment status [15, 16]. This in return affects the feeding practice of children during diarrheal episodes, for instance, mothers who have one under-five child are more likely to have appropriate feeding practices (eating or drinking more) during diarrhea episodes as compared to those who have two or more children [17]. Other factors associated with appropriate feeding practice is women attending antenatal and postnatal visits [18].

Fluid replacement and continued feeding can help control complications and speed up recovery from diarrheal disease. The ability to recognize the factors that influence feeding behavior during a diarrheal episode is a necessary precondition for developing diarrheal disease effective intervention strategies. Although studies were conducted in some parts of the East African countries, no previous study was conducted to estimate eating or drinking more during diarrheal episodes among under-five children using Demographic and Health Surveys (DHS) in East African countries. The previous studies mainly focus on national or subnational level, however currently there is a need of integrating east Africa in different circumstances including the health of the population [19], hence it could give an insight to develop child health programs in a coordinated manner in the region. Therefore this study aimed to estimate the prevalence of drinking or eating more than usual during diarrheal episodes among under-five children in the 12 East Africa Countries from 2008 to 2018 using recent DHS.

## Methods

### Study settings and data source

In this study, the analysis was conducted using secondary data from DHS in east African countries. There are 19 countries in East Africa, of those 13 countries have DHS while six countries did not (Djibouti, Mauritius, Somalia, Somaliland, Seychelles, and Reunion). The study used 12 countries’ (Burundi, Ethiopia, Kenya, Comoros, Rwanda, Mozambique, Tanzania, Madagascar, Zambia, Zimbabwe, Uganda and Malawi), that was conducted after 2008. One country Eritrea was excluded from the study due to restriction of the DHS data. The data were obtained from the official database of the DHS program, www.measuredhs.com after authorization was granted via online request by explaining the purpose of our study. DHS is a nationally representative household survey that collects data on broad range of health indicators like mortality, morbidity, fertility, contraceptive utilization, maternal and child health [20, 21]. In this study, we used the child record datasets (KR file), and extract the outcome and independent variables. The surveys employ stratified, multi-stage, random sampling techniques. Detailed survey techniques and sampling methods used to collect data have been documented somewhere [22].

### Study variables

#### Outcome variable

Feeding practice (drinking or eating more than usual) during childhood diarrhea episodes. The respondents were asked, the amount of foods offered or amount of liquids given during diarrheal episodes 2 weeks prior to the survey in the categories of “more than usual”, “same as usual”, “somewhat less”, “much less”, “none”, “never gave food”, or “don’t know” [20]. Then the outcome is derived after merging thevariable of the amount of food and liquid given and coded 1 if drinking or eating more than usual and the rest coded as 0 [20, 23].

#### Independent variables

The independent variables were categorized into individual-level and community-level variables after a thorough literature review [17, 18, 24, 25]. The individual-level variables are age, level of education, marital status of respondents, number of ANC visits, place of delivery, distance from the health facility, and wealth status, whereas, the community-level variables include community-level poverty, media exposure, and residence which was driven from individual-level variables. The community poverty level was measured using the household wealth index. The proportion of women in households with a low household wealth index was then calculated and classified as low poverty (those with < 50%) and higher poverty (those with > 50%) using the national median value. Community-level media exposure was created from the respondents’ exposure to newspaper/magazine, radio, and television after merging them and recoding them to Yes/No. since the data were not normally distributed, the median was utilized, and the results were classified as low if less than 50% of respondents had exposure to at least one medium, and high if more than 50% of respondents had exposure to at least one medium.

### Data management and statistical analysis

In this study, Stata version 16 software was used for data analysis. Prior to data analysis, the data were weighted to ensure representativeness of the DHS sample and to obtain reliable estimates and standard errors. We applied weighting for sampling weight using women’s individual sampling weight while we run all the analysis. We used cross-tabulations and summary statistics for the descriptive results. Four models were fitted in this study: the null model, which had no explanatory variables, model I, which had individual-level factors, model II, which had community-level factors, and model III, which had both individual and community-level components. Since the models were nested, the Intra-class Correlation Coefficient (ICC), Median Odds Ratio (MOR) and Likelihood Ratio test (LLR), deviance (−2LLR) values were used for model comparison and fitness, respectively. Model III was chosen as the best-fitted model since it had the lowest deviation. Variables having a *p*-value less than 0.2 in bivariable were used for multivariable analysis. Finally, in the multivariable analysis, adjusted odds ratios with 95% confidence intervals and a p-value of less than 0.05 were utilized to identify associated factors of drinking or eating more than usual. Forest plot was used to show the overall and the prevalence of each country.

## Results

### Sociodemographic characteristics

A total of 20,559 mothers/caregivers with their respective under-five children with recent diarrhea were included in the study. Out of all the study participants, 45.59% were between the age of 25–34 years, and the mean age with standard deviation was 27.80 + 6.72. The majority of the participants 16,223(78.91%) were from rural area. From all the countries included 17.43% were from Malawi (Table 1).

**Table 1:** Socio-demographic and other characteristics of respondents in East Africa (*n* = 20,559)

Variables	Category	Frequency	Percent
Age in years	15–24	7452	36.25
	25–34	9374	45.59
	> 35	3733	18.16
Residence	Urban	4336	21.09
	Rural	16,223	78.91
Country	Burundi	2877	13.99
	Comoros	514	2.50
	Ethiopia	1227	5.97
	Kenya	2841	13.82
	Madagascar	993	4.83
	Malawi	3584	17.43
	Mozambique	1204	5.86
	Rwanda	929	4.52
	Tanzania	1122	5.46
	Uganda	2832	13.77
	Zambia	1422	6.92
	Zimbabwe	1014	4.93
Sex of household head	Male	15,609	75.92
	Female	4950	24.08
Current marital status of respondents	Married	13,960	67.90
	Not married	6599	32.10
Educational status of respondents	No education	4598	22.37
	Primary education	11,337	55.14
	Secondary education	4135	20.11
	Higher education	489	2.38
Educational status of partner(*n* = 16,632)	No education	3318	19.95
	Primary education	8519	51.22
	Secondary education	4049	24.34
	Higher education	746	4.49
Occupation of respondents	Not working	5042	24.53
	sales and services	2722	13.24
	agricultural	9474	46.08
	Manual	3262	15.87
	Others	59	0.29
Wealth status	Poor	9803	47.68
	Middle	3940	19.16
	Rich	6816	33.15
Community-level poverty	Low	10,534	51.24
	High	10,025	48.76
Community-level media exposure	Low	10,125	49.25
	High	10,434	50.75
Visit the health facility for the last 12 months (*n* = 19,063)	No	4167	21.86
	Yes	14,896	78.14
Distance to the health facility (*n* = 18,542)	Big problem	8303	44.78
	Not big problem	10,239	55.22

### Prevalence of drinking or eating more than usual during diarrhea episodes among children aged less than 5 years

**Fig. 1:**
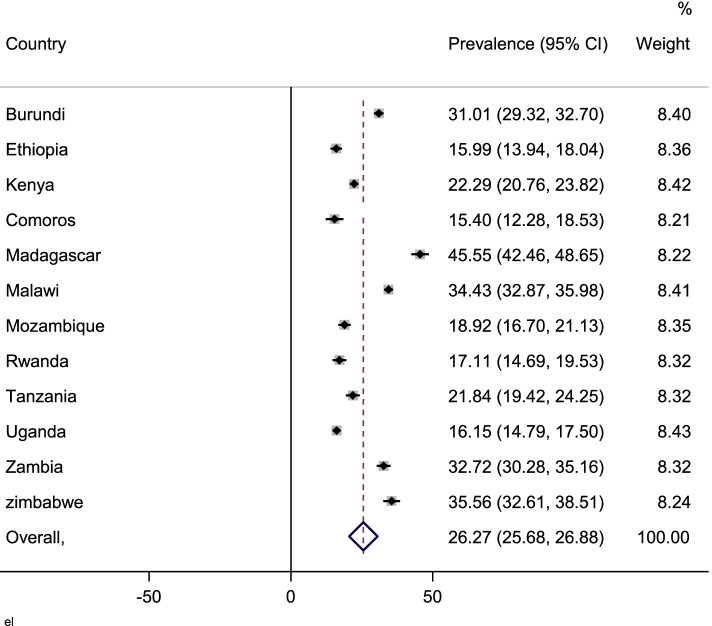
Forest plot of overall prevalence of drinking or eating more than usual in the 12 East Africa Countries from 2008 to 2018

Out of 20,559 children, only 1520 (7.39%) were offered food to eat more than the usual amount, and 5010 (24.37%) to drink more liquids. The prevalence of drinking or eating more than usual during diarrhea disease in East Africa was found to be 26.27% (95% CI: 25.68–26.88). Ranges from 15.40% in Comoros to 45.55% in Madagascar (Fig. 1).

### Obstetrics related characteristics of respondents

The majority of mothers (45.70%) had four or more antenatal care visits during pregnancy. Nearly 60% of the participants give their first birth between the ages of 18–24. Approximately three-fourths (72.23%) of mothers’ place of delivery was at a health facility (Table 2).

**Table 2:** Obstetric-related characteristics of mothers in East Africa (*n* = 20,559)

Variables	Categories	Frequency	Percent
Number of ANC visit	0	3973	19.32
	1–3	7191	34.98
	4+	9395	45.70
Current pregnancy	No	19,006	92.45
	Yes	1553	7.55
Number of living children	1	4684	22.78
	2–5	12,986	63.17
	6+	2889	14.05
Age of mother at first child in years	< 18	6892	33.52
	18–24	12,301	59.83
	25+	1366	6.64
Place of delivery (*n* = 20,539)	Home	5708	27.77
	Health facility	14,851	72.23

### Child characteristics and common childhood illnesses

A majority of the children (52.77%) were male,53.06% were between the ages of 1 and 2 months. The majority of the children (61.50%) were currently breastfed. Regarding the symptoms, 45.97% had a fever and 44.30% had a cough in the 2 weeks preceding the survey, respectively (Table 3).

**Table 3:** Child characteristics and common childhood illness in East Africa (*n* = 20,559)

Variables	Category	Frequency	Percent
Sex of child	Male	10,848	52.77
	Female	9711	47.23
Current age in years	< 1	5432	26.42
	1–2	10,908	53.06
	3+	4219	20.52
Child is twin	Single	20,022	97.39
	Multiple	537	2.61
Currently breast feed	No	7915	38.50
	Yes	12,644	61.50
Had fever in the last two weeks (*n* = 20,541)	No	11,099	54.03
	Yes	9442	45.97
Had cough in the last two weeks (*n* = 20,532	No	11,436	55.70
	Yes	9097	44.30

### Factors associated with drinking or eating more than usual during diarrheal episodes

In this analysis, the multivariable multilevel model was fitted and found ICC of 6.8% (95%.

CI: 5.5, 8.3) and deviance of 21,456.484. From the final model age of respondents, occupation,, educational status, marital status, number of antenatal care visits, place of delivery, visiting health facility in the past 12 months, wealth status and child age were variables significantly associated with drinking or eating more than usual during diarrhea for children aged less than 5 years. Accordingly, children whose mother is in the age group of > 35 years increases the odds of eating or drinking more than the usual during diarrheal episodes by 1.14 times (AOR = 1.14, CI: 1.03, 1.26) compared to mothers with the age group of 15–24 years. Children whose mothers had primary education, secondary education, higher education 1.17 (AOR: 1.17, 95% CI: 1.06, 1.28), 1.43 (AOR: 1.43, 95% CI: 1.27, 1.61), 1.42(AOR: 1.42, 95% CI: 1.11, 1.81) times more likely to drink or eat more than usual compared to mothers with no education, respectively. Children whose mother is married were 1.27 times more likely to drink or eat more than usual when compared with unmarried ones (95% CI: 1.18, 1.37). Children whose mother works in sales and services, agriculture occupations, and manual work, 1.20 (AOR = 1.20, CI: 1.07, 1.34), 1.36 (AOR = 1.36, CI: 1.24, 1.47), and 1.28 (AOR =1.28, 95% CI: 1.11, 1.44) times more likely to drink or eat more than usual compared to mothers with no work, respectively. Children from the middle family 1.13 (AOR =1.13, 95% CI: 1.03, 1.23) and the rich family 1.27(AOR =1.27, 95%CI: 1.16, 1.40) times more likely to drink or eat more than usual compared with poor families. Children being in the age group of 1–2 years (AOR =1.34, 95% CI: 1.22, 1.46) and 3 and above years (AOR =1.36, 95% CI: 1.20, 1.55) are more likely to drink or eat more than usual compared with infants. Children whose mothers attended four or more antenatal visit (AOR: 1.14, 95% CI: 1.03, 1.27) were more likely to drink or eat more than usual compared with no visits. Mothers who gave birth at a health facility (AOR = 1.19, 95%CI: 1.10, 1.30) were more likely to give more drink or food to their children. Mothers who visit health facilities in the last 12 months (AOR = 1.12, 95%CI: 1.03, 1.22) were more likely to give more fluid or food to their children.

In the empty model, the values of Intra Class Correlation (ICC = 7.66%) and Median Odds Ratio (MOR = 1.64) implies the presence of community-level variability of drinking or eating more. Around 8% of the variation in modern contraceptive use is attributed to ICC. In the empty model, the presence of heterogeneity of drinking or eating more between clusters is indicated by the MOR with a value of 1.64. It indicates that if we randomly select under-five age children, a child at the cluster with higher drinking or eating had around 1.64 times higher odds of drinking or eating more than a child at cluster with lower drinking or eating. Model III had the lowest deviance value (21,456.484) and hence it was selected as the best-fitted model (Table 4).

**Table 4:** Factors associated with drinking or eating more than usual during diarrheal episodes in East Africa (*n* = 20,559)

Variables	Null model	Model I AOR (95% CI)	Model II AOR (95% CI)	Model III AOR (95% CI)
**Age of respondents**				
15–24		1		1
25–34		0.99(0.92,1.07)		0.99(0.92,1.07)
> 35		1.14(1.03, 1.26)		1.14(1.03, 1.26)*
**Residence**				
Rural			1	
Urban			1.21(1.11,1.32)	0.97 (0.87,1.08)
**Marital status**				
Not married		1		1
Married		1.27(1.18,1.37)		1.27(1.18,1.37)***
**Educational status**				
No education		1		1
Primary education		1.16(1.06,1.27)		1.17(1.06,1.28)**
Secondary education		1.42(1.27,1.60)		1.43(1.27,1.61)**
Higher education		1.40(1.10,1.79)		1.42(1.11,1.81)**
**Occupation of respondents**				
Not working		1		1
Sales and services		1.19(1.06, 1.34)		1.20(1.07,1.34)**
Agricultural		1.36(1.25,1.49)		1.36(1.24,1.47)***
Manual		1.27(1.11,1.44)		1.28(1.11,1.44)***
Others		1.20(0.65,2.18)		1.18(0.65,2.16)
**Wealth status**				
Poor		1		1
Middle		1.14(1.04, 1.25)		1.13(1.03 1.23)*
Rich		1,29(1.18, 1.40)		1.27(1.16, 1.40)***
**Community-level poverty**				
High			1	
Low			1.18(1.07,1.32)	1.10(0.99, 1.22)
**Number of ANC visit**				
0		1		1
1–3		1.06(0.95,1.18)		1.06(0.95,1.18)
4+		1.14(1.03,1.27)		1.14(1.03,1.27)*
**Place of delivery**				
Home		1		1
Health facility		1.19(1.10,1.30)		1.19(1.10,1.30)***
**Child age**				
< 1 year		1		1
1–2 years		1.34(1.22,1.46)		1.34(1.22,1.46)**
3–4 years		1.36(1.20,1.55)		1.36(1.20,1.55)**
**Currently breastfeeding**				
No		1		1
Yes		0.98(0.84,1.00)		0.92(0.83,1.00)
**Community-level media exposure**				
Low			1	
High			0.95(0.86,1.05)	0.92 (0.83,1.02)
**Visit the health facility for the last 12 months**				
No		1		1
Yes		1.12(1.03,1.22)		1.12(1.03,1.22)**
Intercept	0.27(0.22,0.34)	0.11(0.09,0.14)	0.29(0.2,0.32)	0.11(0.09,0.14)
**Measure of variations**				
Log-likelihood	−11,688.45	−10,730.50	−11,670.46	−10,728.24
Deviance	23,376.89	21,461.01	23,340.91	21,456.48
Variance	0.27	0.24	0.26	0.23
ICC	7.67(6.32, 9.26)	6.83(5.54,8.34)	7.54(6.21,9.12)	6.80(5.53,8.33)
PCV	Reference	11.1%	3.7%	14.8%
MOR	1.64	1.59	1.62	1.57

Null model-contains no explanatory variables; Model I-includes individual-level factors only; Model II-includes community-level factors only; Model III includes both individual-level and community-level factors, *AOR* adjusted odds ratio, *CI* confidence internal, *ICC* intraclass correlation coefficient, *MOR* median odds ratio, *PVC* proportional change in variance.

*** P-value < 0.001, ** p-value < 0.01, * p-value < 0.05

## Discussion

Mothers or caregivers are recommended to enhance feeding practices, notably giving children more fluid and food than normal, to avoid mortality, dehydration, and the consequences of diarrhea on nutritional status [10, 26, 27]. Oral rehydration therapy is the most commonly prescribed treatment for diarrhea. Rice water, yogurt, soup, salt sugar solution, and clean water are some home-based fluids that are recommended [28]. The findings of our study will help policy and program makers to develop tailored intervention strategies by considering the level of feeding practice and the factors associated with it.

In this study, the overall prevalence of drinking or eating more than usual during diarrheal episodes was 26.27%(95% CI: 25.68–26.88), and maternal age, mother’s occupation, antenatal care visit, marital status, educational status, wealth status, place of delivery, child age, and visiting health facility in the past 12 months were significantly associated with drinking or eating more than usual during diarrheal episodes.

This study showed that the prevalence of drinking or eating more than usual during diarrheal episodes was similar to a study conducted in Karachi, Pakistan (26.2%) [29]. It is higher than a study done in Ethiopia DHS (15.4%) [18]. This variation may be due to the difference in sociocultural and socioeconomic status. However, the prevalence of drinking or eating more than usual in this study is lower than a study conducted in Ethiopia, West Hararge Zone (40.8%), in Burayu Town (53.6%) [30], and Gamo Gofa zone (70.7%) [17]. This may be due to the fact that nationally representative data in our study and the difference in the study populations (those studies recruited less than 2 years).

The odds of drinking or eating more than usual among children whose mothers aged > 35 years were higher compared to mothers whose age is between 15 and 24 years. This was supported in a study done in Ethiopia [17, 31], and a study done in Nellore District, South India [24] where mothers’ age is associated with good knowledge to feed their children. The possible reason could be as a woman gets older the probability of getting information about feeding practice and learning from their experience is getting higher and higher. The other possible reason might be older women exposed to health-related information during their consecutive pregnancy visits.

This study showed that the odds of drinking or eating more than usual among children whose mother married was higher compared with unmarried mothers. The possible reason might be those married mothers may have better coordination with their spouse, the increase in activity to the household and they may get better knowledge from their parents. Parents’ support are essential for the health of their children [32].

Children whose mothers education primary, secondary, and higher education have higher odds of drinking or eating more than usual compared with those who have no education. This was supported by a study done in Ugandan children where maternal literacy is associated with better infant and young children feeding practice [33]. This is in fact, health knowledge and behavior are improved by education, the higher the education the ability to read and comprehend the nutrition requirement guideline increases [34, 35]. In addition, access and ability to search health related information increases. This implies that educating mothers could be one strategy to improve children feeding practice during diarrheal episodes.

The odds of drinking or eating more than usual among children whose mothers worked in sales and services, agriculture and manual were higher compared with children whose mothers did not have work. Participating in work may expose mothers to information regarding feeding practices from peers and friends [29, 36, 37].

This study revealed that the odds of drinking or eating more than usual among children with middle and rich families were higher compared with the poor ones. The possible reason could be children from the poor household have poor access to adequate food, which makes them less likely to eat or drink more than the usual amount [38–41]. It means that being wealthiest determine the ease of accessing resources to meet one’s own need and in return children’s feeding practice during diarrheal episodes. This implies that there is a need to improve household wealth status.

The results of this study show that the odds of drinking or eating more than usual among children aged above 1 year is higher compared with infants. This was supported by different studies which reported that children aged between 6 and 11 months were not given appropriate complementary feeding [42, 43]. This could be due to the reason the practice of complementary feeding as infants are less likely to eat food as compared with older children. This implies that there is a need for health programme intervention to pay more attention to give more fluids or foods during the diarrheal episode for infants, hence they are at risk of fluid loss compared with older children [44].

The current study showed that mothers who had ANC visits, who gave birth at health facility, and who visit health facilities in the last 12 months, were more likely to drink or eat more than usual. This is consistent with studies which show mothers who had more than four ANC visits feed their children appropriately [17, 29]. This might be due to improved access to health-care facilities increases the amount of health-related information received from health workers, including information on how to feed during diarrheal episodes. These facilities are a good place to go for advice on child feeding practices, as well as maternal and child care. Health professionals may provide information, education, and counseling to mothers who had ANC and who gave birth at health facilities, and who visit health facilities about proper child feeding practices for normal child growth and during an illness such as diarrhea disease.

### Strengths and limitations of the study

Regarding the strengths, the study uses large data set from 12 east African countries, which is representative across the countries. This study also used a multilevel modeling technique to come up with a more reliable result that takes into account the survey data’s hierarchical nature. However, the study is not free of limitations, the survey is prone to social desirability due to the self-reported nature of the interview, and the cross-sectional nature of the study may not explain the temporal relationship of the independent and the outcome variables.

## Conclusions

This study shows that drinking or eating more than usual during diarrheal episodes is low in East African countries. Maternal age, mothers occupation, antenatal care visit, marital status, educational status, wealth status, place of delivery, visiting health facility in the past 12 months, and child age were significantly associated with drinking or eating more than usual during diarrheal episodes. Health policy and programs should focus on educating mothers/caregivers, improving the wealth status, encouraging women to contact health facilities for better feeding practice of children during diarrheal episodes.

## Data Availability

The availability of the data set for this study was obtained from the DHS program data sets using the website www.measuredhs.com after we have sent research objectives.

## Abbreviations

AOR: Adjusted Odds Ratio
DHS: Demographic Health Survey
ICC: Intra-class Correlation Coefficient
MOR: Median Odds Ratio
PCV: Proportional Change in Variance
SD: Standard Deviation
WHO: World Health Organization
